# The effect of CHIN-SKIP sports game intervention on motor ability in preschool children aged 5–6 years

**DOI:** 10.3389/fpubh.2025.1643521

**Published:** 2025-08-22

**Authors:** Shiqing Zhang, Min Liu, Jianhang Sun, Jianxiang Chong, Jiahua Ma

**Affiliations:** Physical Education Institute, Shanxi University, Taiyuan, China

**Keywords:** CHIN-SKIP, motor ability, preschool children, sports game intervention, aged 5–6 years

## Abstract

**Introduction:**

To evaluate the efficacy of CHIN-SKIP sports game intervention in enhancing motor ability among preschool children aged 5–6 years.

**Methods:**

From September to December 2024, a total of 60 preschool children (aged 5–6 years) were randomly selected from two large classes at DM kindergarten and equally allocated to either the experimental group (*n* = 30) or control group (*n* = 30), with balanced gender distribution between groups. The experimental group received the CHIN-SKIP sports game intervention, while the control group participated in standard kindergarten physical activities. The 12-week intervention consisted of two 30-min sessions per week. Motor ability was assessed pre- and post-intervention using the “Manual for Testing Motor Ability in Children Aged 3–6 Years” developed by the China Institute of Sports Science (CISS), which evaluates five distinct domains of motor ability.

**Results:**

Mixed ANOVA revealed significant main effects of both group assignment and time on motor ability outcomes (all *p* < 0.01). The experimental group demonstrated superior performance in total motor quotient (TMQ) and gross motor quotient (GMQ) compared to controls (*p* < 0.01). Temporal improvements were observed across all measures (*p* < 0.01), with TMQ and GMQ showing the most pronounced enhancements. Significant group × time interactions (*p* < 0.001) indicated greater improvement in the experimental group for all measures except visual-motor integration.

**Conclusion:**

The CHIN-SKIP sports game intervention effectively enhances motor ability in 5-6-year-old preschool children and shows promise for broader implementation in preschool children education settings, thereby establishing a strong foundation for developmental progress.

## 1 Introduction

Motor ability is the capacity of the body to perform in motion (1), which is specifically divided into gross and fine motor ability. The age of 3–6 years is a critical period for children’s physical and mental development, as well as a “window period” for the formation and development of motor ability (2). The model of children’s motor ability and physical activity development trajectory developed by Professor Stodden et al. (3) from the Department of Physical Education at the University of South Carolina in the United States posits that motor ability serves as a primary intrinsic determinant of physical activity behaviors throughout childhood and adolescence (4).

Recent data from China’s Fifth National Physical Fitness Monitoring Bulletin reveal concerning trends while anthropometric measures (height, weight, sitting height) have improved among 3–6-year-olds since 2014, most physical fitness parameters have declined significantly, with balance beam performance being the sole exception. Concurrent national surveys indicate that only 62.3% of preschool children meet the recommended daily physical activity guideline of ≥60 min, as established by China’s Kindergarten Work Regulations and Childhood Obesity Prevention Guidelines. This physical activity standard encompasses structured movement exercises including locomotor activities (running, jumping), sports games, and cycling. Notably, over one-third of Chinese preschoolers fail to achieve this benchmark, highlighting an urgent need for targeted motor skill interventions.

The CHIN-SKIP sport game intervention, developed by Dr. Tao Xiaojuan (23), represents an innovative adaptation of the evidence-based SKIP (“Successful Kinesthetic Instruction for Preschoolers”) framework (5). This localized intervention incorporates three key theoretical foundations: dynamic systems theory, Newell’s constraints model, and Metzler’s instructional design framework. The program features several distinctive elements: (1) a gamified curriculum structure with embedded feedback mechanisms to enhance skill acquisition; (2) a three-tiered physical literacy framework addressing limitations in original SKIP program objectives; (3) developmentally appropriate content sequencing across preschool grade levels; (4) cultural adaptations including traditional festival themes and teacher-directed implementation. Indonesian researchers Bakhtiar et al. (6) developed “INDO-SKIP,” a motor skill-based intervention program specifically designed for Indonesian kindergartens. Subsequent implementation studies by Famelia et al. (7) demonstrated INDO-SKIP effectively enhanced motor ability, perceptual-motor skills, and executive function in young Muslim children in Indonesia. Furthermore, empirical evidence has established SKIP’s significant efficacy in addressing developmental motor delays in children (8). Based on this, the present study hypothesizes that CHIN-SKIP sports game intervention can more effectively enhance the motor ability of preschool children aged 5–6 years old compared with regular activities in kindergarten.

## 2 Method

### 2.1 Participants

Through lottery, 60 preschool children aged 5–6 years old in two large classes were randomly selected from the DM kindergarten cluster, with 30 participants in each class. Gender distribution was balanced between groups. Participants were then randomly assigned to either the experimental (*n* = 30) or control (*n* = 30) group. Inclusion Criteria: (1) Typically developing intelligence, vision, and hearing; (2) Age 5–6 years old; (3) Capacity to participate in standard sports games; (4) Provision of informed consent by legal guardian (written consent). Exclusion Criteria: (1) Atypical physical development; (2) Congenital disorders or chronic medical conditions; (3) Participation in structured extracurricular sports programs. In this study, the personal information of the subjects is kept absolutely confidential, and the parents of the subjects have given informed consent to the process of this trial and the possible dangers, and have signed the informed consent form. The study was approved by the Shanxi University Ethics Committee (No. SXULL2024082).

### 2.2 Method

#### 2.2.1 Experimental design

The study was conducted from September to December 2024, comprising four distinct phases: pre-experiment, baseline assessment, intervention implementation, and post-intervention evaluation. Six graduate students in physical education from Shanxi University were recruited and assigned to either teaching (*n* = 3) or assessment (*n* = 3) roles. Assessors maintained blinding by remaining unaware of group assignments throughout the study. All personnel completed 72 h of standardized training in motor development assessment under the supervision of expert instructors prior to data collection.

A one-week pre-experimental period was conducted to achieve three primary objectives: ensure instructor proficiency with testing protocols, validate the methodological soundness of the assessment design, and implement necessary modifications to the testing regimen if required. The baseline assessment mainly evaluates the basic condition and motor ability of the subject. The 12-week intervention consisted of two 30-min sessions weekly. The control group followed standard kindergarten physical activities, while the experimental group received the structured CHIN-SKIP curriculum. Post-intervention assessments, identical to baseline measures in methodology, personnel, and instrumentation, were completed within 1 week following intervention cessation. The complete study timeline spanned 15 weeks.

#### 2.2.2 Training protocol

As detailed in Table 1, the control group’s sessions followed a standardized three-phase structure: (1) an initial phase (5 min) involving class organization, curriculum introduction, and warm-up exercises; (2) a core instructional phase (21 min) comprising skill demonstrations, game rules, and safety monitoring; and (3) a concluding phase (4 min) with guided stretching and session debriefing. The experimental group maintained this structure but incorporated a specialized rotation system during the core phase, featuring three motor skill stations arranged in counterbalanced sequences (1 → 2 → 3, 2 → 3 → 1, 3 → 1 → 2) to enhance skill acquisition, engagement, and structural flexibility while ensuring developmental appropriateness and school transition readiness. Detailed curriculum content and implementation protocols for the experimental group are provided in Tables 2, 3.

**Table 1:** Instructional framework for control group sessions.

Session component	Detailed protocol	Duration
Initial phase	1. Class organization and attendance	5 min
	2. Lesson objectives and content introduction	
	3. Music-guided warm-up activities	
Core instructional phase	1. Developmentally appropriate activities implementation	21 min
	2. Movement technique demonstration and safety protocols	
	3. Game rule explanation and classroom management	
Concluding phase	1. Supervised cool-down exercises with music	4 min
	2. Session debriefing and equipment collection	

**Table 2:** CHIN-SKIP sports game intervention curriculum structure for the experimental group.

Session	Skill-1	Skill-2	Skill-3
1	Unilateral hopping	Bilateral consecutive jumping	Ball rolling
2	High jumping	Ball catching	Running
3	Stride jumping	Postural control (center of gravity shift)	Cardiorespiratory endurance
4	Running	Manual ball dribbling	Balance
5	Running	Bilateral ball striking	Load
6	High jumping	Cardiorespiratory endurance	Postural control (center of gravity shift)
7	Unilateral hopping	Stride jumping	Muscular strength
8	Bilateral ball striking	Horse running	Controlled landing
9	Lateral sliding	Ball kicking	Cardiorespiratory endurance
10	Unilateral hopping	Foot dribble	Muscular strength
11	Crawl	High jumping	Load
12	Bilateral consecutive jumping	Horse running	Balance
13	Ball catching	Crawl	Flexibility
14	Long jumping	Load	Muscular strength
15	Running	Ball catching	Balance
16	Ball rolling	Flexibility	Load
17	Controlled landing	Throwing the ball	High jumping
18	Running	Straddle	Manual ball dribbling
19	Long jumping	Foot dribble	Lateral sliding
20	Ball kicking	Throwing the ball	Flexibility
21	Lateral sliding	Bilateral ball striking	Muscular strength
22	Ball catching	Straddle	Postural control
23	Horse running	Controlled landing	Muscular strength
24	Running	High jumping	Straddle

**Table 3:** Case study of the organization and implementation of the course tasks of the experimental group.

Duration	Organization	Implement
1 min Introduction	* Preschool children sit around the circular marker * The teacher plays a role based on the theme of the game and announces the lesson theme and task * The teacher explains the tasks of the three skill stations	Equipment: A certain number of markers; Safety tip: Walk toward the marker, find it and sit down immediately, do not scramble.
2 min Warm-up	* Children stand at the circular marker and practice their motor skills with music under the guidance of the teacher	Equipment: Tape recorders, marker points, etc.
7 min Station 1	Teacher 1: * Explain the tasks and requirements in the context of the game theme and present them Toddler: * Stand on the sign point and stay focused *Complete the skill exercises and pick up the equipment and put it back in place *After completing multiple exercises in order, assist your companion in putting all the equipment back in place before turning to the next station	Equipment: The required sports equipment, items required for environmental layout, etc., the quantity is determined by the number of children; tips for key elements of motor skills; Observe the child’s motor skills
7 min Station 2	Teacher 2: * Explain the task requirements and present them in combination with the theme of the game; Toddler: * Stand on the sign and listen intently * Practice motor skills; * Challenge: Count the number of times you or your companions have successfully completed a skill	
7 min Station 3	Teacher 3: * Explain task requirements and demonstrate motor skills, organize the application and combination of motor skills Toddler: *Stand on the marker and listen to the teacher’s explanation * Play the game; take back the equipment	
5 min Cool-down	Teacher: * Lead the children to carry out ontological relaxation activities, and organize the sharing, feedback and summary of the class, and give children rewards; Toddler: * Return to the original warm-up area, sit around, relax, and share;	Equipment: Before the child returns, form a circle around the marker; classroom prizes, such as stickers, toys, etc.
1 min Closure	Teacher: *With the assistance of the classroom teacher, organize the children to stand at the beginning of the class in order Toddlers: *Return to the starting point and leave the playing field	Equipment: Teachers collect and organize

### 2.3 Outcome measures

#### 2.3.1 Test content

The motor ability assessment was conducted using the “Manual of Motor Ability Testing for Children Aged 3–6 Years” developed by the CISS, which adapts items from the Peabody Motor Development Scale. Although the manual’s psychometric properties have not been formally published in peer-reviewed journals, the CISS-issued manual is recognized as authoritative, and the Chinese version of the Peabody scale has demonstrated good reliability and validity in previous studies with Chinese children (9). The assessment comprised five domains: (1) Posture: Evaluating static balance and body control through one-leg standing, tiptoe standing, and sit-ups; (2) Movement: Assessing movement between marked locations via backward walking, straight-line walking, and stair navigation; (3) Physical operation: Measuring ball manipulation skills including throwing, bouncing, and catching; (4) Grasping: Testing hand function and grasping ability; (5) Visual-motor integration: Evaluating hand-eye coordination through bead threading and figure copying tasks. The first three domains constituted the Gross Motor Quotient (GMQ), while the latter two formed the Fine Motor Quotient (FMQ). The Total Motor Quotient (TMQ = GMQ + FMQ) served as the primary outcome measure. Most test items were objectively scored (timed performance, distances, counts), with some requiring subjective ratings by trained assessors.

#### 2.3.2 Grading criteria

All assessors completed a 72-h standardized training program to ensure consistent administration and scoring procedures. Inter-rater reliability exceeded 95% agreement on practice items prior to data collection. Scoring criteria were: two points for perfect execution matching standardized demonstrations; 1 point for partial completion with observable errors; and 0 points for inability to attempt or complete the task. Testing continued until either: (a) the child achieved a perfect score, or (b) three attempts were completed, whichever occurred first.

#### 2.3.3 Test equipment

Required equipment and quantities are detailed in Table 4.

**Table 4:** Equipment and quantity required for the test.

The name of the device	Quantity
Yoga mat	1–2
Scotch tape	1–2
Staircase	There must be at least six stairs near the testing site
40–52.5 cm high stable support	1
45–60 cm high stable support	1
Large tape measure	1–2
Chair (height > 25 cm, rope can be tied at 25 cm)	2
90 cm long string or thick rope	1
Empty cans (or anything like a sign bucket, smaller, one that the child can pick up from the ground and carry to run)	1
Marker pen (cylindrical, e.g., black marker)	2–3
White paper (21 × 28 cm, similar in size to A4 paper, can be used directly with A4 paper)	100
Stopwatch	1
Bottles with screw caps	1
Food pills that can be put in from the above bottle	100
Blunt-tipped scissors	2–3
Tennis	1

#### 2.3.4 Testing considerations

Three key protocols ensured assessment validity: (1) Testing occurred in quiet, distraction-free environments; (2) Assessors maintained participant engagement through verbal encouragement and sticker rewards, with breaks scheduled as needed; (3) Strict independence was maintained—no physical or verbal guidance was permitted during task execution.

#### 2.3.5 Statistical methods

Analyses were conducted using SPSS 27.0 software. After confirming normality (Shapiro–Wilk test) and homogeneity of variance (Levene’s test), we employed mixed-design ANOVA with time (pre/post) and group (experimental/control) as factors. Effect sizes were assessed using partial ETA squared. *Post hoc* analysis was performed using the Bonferroni test.

## 3 Results

A mixed-design ANOVA was conducted to examine the effects of group assignment (experimental vs. control) and assessment timepoint (pre- vs. post-intervention) on multiple motor ability indicators, including posture, movement, physical operation, grasping, visual-motor integration, GMQ, FMQ, and TMQ. The analysis revealed significant main effects of time and group, as well as significant group × time interactions across most measures (see Table 5 for complete results).

**Table 5:** Mixed ANOVA results.

Project	Group main effect	Time main effect	Group × Time interaction
Posture	*F* = 3.945, *p* = 0.052, *η*^2^ = 0.067	*F* = 77.917, *p* < 0.001, *η*^2^ = 0.586	*F* = 60.121, *p* < 0.001, *η*^2^ = 0.522
Movement	*F* = 4.163, *p* = 0.046, *η*^2^ = 0.070	*F* = 115.523,*p* < 0.001,*η*^2^ = 0.677	*F* = 76.874, *p* < 0.001, *η*^2^ = 0.583
Physical operation	*F* = 6.020, *p* = 0.017, *η*^2^ = 0.099	*F* = 76.774, *p* < 0.001, *η*^2^ = 0.583	*F* = 47.841, *p* < 0.001, *η*^2^ = 0.465
Grasping	*F* = 8.938, *p* = 0.004, *η*^2^ = 0.140	*F* = 95.490, *p* < 0.001, *η*^2^ = 0.635	*F* = 70.991, *p* < 0.001, *η*^2^ = 0.563
Visual-motor integration	*F* = 0.032, *p* = 0.859, *η*^2^ = 0.001	*F* = 7.955, *p* = 0.007, *η*^2^ = 0.126	*F* = 0.002, *p* = 0.961, *η*^2^ = 0.000
GMQ	*F* = 13.346, *p* < 0.001, *η*^2^ = 0.195	*F* = 240.207, *p* < 0.001, *η*^2^ = 0.814	*F* = 165.385, *p* < 0.001, *η*^2^ = 0.750
FMQ	*F* = 5.444, *p* = 0.023, *η*^2^ = 0.090	*F* = 99.508, *p* < 0.001, *η*^2^ = 0.644	*F* = 54.383, *p* < 0.001, *η*^2^ = 0.497
TMQ	*F* = 19.609, *p* < 0.001, *η*^2^ = 0.263	*F* = 321.993, *p* < 0.001, *η*^2^ = 0.854	*F* = 203.658, *p* < 0.001, *η*^2^ = 0.787

### 3.1 Group main effect

The between-group analysis revealed significant differences in motor ability outcomes across most measures (all *p* < 0.01), with the exception of visual-motor integration (*F* = 0.032, *p* = 0.859, *η*^2^ = 0.001). Most notably, the experimental group demonstrated superior performance in TMQ (*F* = 19.609, *p* < 0.001, *η*^2^ = 0.263) and GMQ (*F* = 13.346, *p* < 0.001, *η*^2^ = 0.195), indicating robust intervention effects on both comprehensive and fundamental motor skills. Significant group differences were also observed in grasping (*F* = 8.938, *p* = 0.004, *η*^2^ = 0.140), while posture approached but did not reach conventional significance levels (*F* = 3.945, *p* = 0.052, *η*^2^ = 0.067), potentially suggesting a modest intervention effect requiring further investigation.

### 3.2 Time main effect

The time main effect demonstrated significant improvements in all motor ability measures between assessment time points (all *p* < 0.01). The most substantial changes were observed in TMQ (*F* = 321.993, *p* < 0.001, *η*^2^ = 0.854), GMQ (*F* = 240.207, *p* < 0.001, *η*^2^ = 0.814), and movement (*F* = 115.523, *p* < 0.001, *η*^2^ = 0.677), indicating particularly robust developmental gains in these domains. Significant temporal improvements were also evident in FMQ (*F* = 99.508, *p* < 0.001, *η*^2^ = 0.644), grasping (*F* = 95.490, *p* < 0.001, *η*^2^ = 0.635), and physical operation (*F* = 76.774, *p* < 0.001, *η*^2^ = 0.583). While visual-motor integration showed more modest but still statistically significant improvement (*F* = 7.955, *p* = 0.007, *η*^2^ = 0.126), its developmental trajectory was less pronounced than other measures.

### 3.3 Group × time interaction

Significant interaction effects emerged for all measures except visual-motor integration (*F* = 0.002, *p* = 0.961, *η*^2^ = 0.000), revealing differential developmental patterns between experimental and control groups. The strongest interactions occurred for TMQ (*F* = 203.658, *p* < 0.001, *η*^2^ = 0.787), GMQ (*F* = 165.385, *p* < 0.001, *η*^2^ = 0.750), and movement (*F* = 76.874, *p* < 0.001, *η*^2^ = 0.583), indicating the intervention’s pronounced effect on these domains. Significant interactions were also observed for FMQ (*F* = 54.383, *p* < 0.001, *η*^2^ = 0.497) and grasping (*F* = 70.991, *p* < 0.001, *η*^2^ = 0.563), demonstrating group-specific developmental trajectories in fine motor abilities.

### 3.4 Multiple correction analysis

To account for multiple comparisons, we applied Bonferroni correction by adjusting the significance threshold (*α*) to 0.0021 (original *α* = 0.05 divided by 24 total comparisons: eight measures × three effects). Post-correction analysis revealed: (1) all temporal main effects and interaction effects remained statistically significant; (2) among group main effects, only grasping, GMQ, and TMQ retained significance after correction; and (3) visual-motor integration showed no significant effects at either the corrected or uncorrected threshold.

## 4 Discussion

This study employed mixed ANOVA to evaluate the efficacy of CHIN-SKIP, a structured sports game intervention, demonstrating significant improvements in children’s motor abilities compared to conventional activities. The intervention’s effectiveness appears attributable to two key design elements: (1) Structured skill stations that provide clear developmental objectives through systematic rotation, addressing the goal ambiguity inherent in traditional game-based teaching while maintaining engagement; (2) Gamified activities that align with children’s natural play tendencies, embedding skill development within an enjoyable framework. This “play-embedded learning” approach successfully balances targeted skill acquisition with the inherent enjoyment of physical play, offering a novel pedagogical model for movement education.

Notably, the study revealed non-significant improvements in visual-motor integration, highlighting current intervention limitations. Several plausible explanations emerge for this finding: First, as a higher-order motor skill, visual-motor integration development relies more heavily on cognitive maturation than basic muscular training. Second, the design of the experimented kindergarten recess exercises is mainly based on simple and repetitive upper limb movements, and lacks complex action tasks that require visual guidance, resulting in the ability is not fully stimulated and developed. Third, visual-motor integration demonstrates particular sensitivity to specific training methodologies, requiring targeted exercises (e.g., drawing tasks) designed explicitly to enhance eye-hand coordination.

Current domestic movement interventions for children exhibit a predominant focus on gross motor development, with comparatively limited emphasis on fine motor skill acquisition, typically employing simplistic instructional designs rather than comprehensive curricular approaches. In contrast, internationally established programs like SPARK and CHAMPPS demonstrate more systematic, developmentally sequenced methodologies. Given early childhood’s critical window for physical and cognitive development (9–12), research confirms well-designed movement programs yield multifaceted benefits including enhanced motor competencies (13–15, 24, 25), social-emotional development (16, 17), cognitive functioning (18–20), and learning strategies (19). Motor development is influenced by three key factors: (1) individual characteristics (age, innate ability) (21), (2) social determinants (parental support) (22), and (3) environmental resources (26, 27), which collectively shape long-term motor competence with downstream effects on physical activity levels, body awareness, and metabolic health. These findings underscore the imperative for educators and caregivers to implement developmentally appropriate movement experiences during this formative period.

## 5 Limitations

Several limitations should be acknowledged: (1) The modest sample size may limit the generalizability of findings, warranting larger-scale replications to validate CHIN-SKIP’s efficacy; (2) The 12-week intervention period precludes assessment of long-term effects, suggesting need for longitudinal follow-up studies; (3) Potential confounding variables (e.g., familial socioeconomic status, genetic predispositions) were not systematically controlled, which future research should address.

## 6 Conclusion

Preliminary evidence from localized studies demonstrates that the CHIN-SKIP sport game intervention significantly enhances motor ability in 5-6-year-old preschool children, though caution is warranted when generalizing these findings due to sample size and geographical limitations. For practical implementation, teachers should receive systematic training in skill station rotation and movement assessment before incorporating CHIN-SKIP into weekly curricula, supported by detailed instructional packages tailored to school-specific conditions. A phased, evidence-based rollout—beginning with pilot testing across diverse kindergarten settings (urban/rural), followed by comparative effectiveness analysis—is recommended to establish guidelines for broader dissemination, ultimately addressing gaps in preschool motor development through scalable, empirically validated strategies. From the perspective of practical application, this study proposes a multi-tiered implementation strategy for integrating CHIN-SKIP into preschool physical education programs. At the instructional level, teachers should incorporate CHIN-SKIP into weekly curricula following systematic training in skill station rotation protocols and standardized movement assessment to ensure intervention fidelity. Concurrently, comprehensive teaching packages—including detailed lesson plans and equipment specifications—must be developed to support consistent implementation. At the institutional level, kindergartens should conduct pilot programs tailored to their student populations and facility conditions, systematically monitoring children’s motor proficiency gains and teacher feedback to iteratively refine the approach. For policy implementation, education authorities should initiate comparative effectiveness trials across diverse settings (urban/rural), using empirical findings to develop evidence-based scaling guidelines. This structured framework addresses preschool motor competency deficits while establishing a rigorous foundation for large-scale dissemination through data-driven adaptation.

## Data Availability

The original contributions presented in the study are included in the article/supplementary material, further inquiries can be directed to the corresponding author.
